# Fuzheng Quxie Decoction Ameliorates Learning and Memory Impairment in SAMP8 Mice by Decreasing Tau Hyperphosphorylation

**DOI:** 10.1155/2017/5934254

**Published:** 2017-12-20

**Authors:** Yang Yang, Xingxing Jia, Jianchao Feng, Zhiyong Wang, Yu Cao, Jiangang Liu, Hao Li

**Affiliations:** ^1^Xiyuan Hospital, China Academy of Chinese Medical Sciences, Beijing 100091, China; ^2^Graduate School, Beijing University of Chinese Medicine, Beijing 100029, China; ^3^Datong Hospital of Traditional Chinese Medicine, Shanxi 037004, China; ^4^Heze Hospital of Traditional Chinese Medicine, Shandong 274002, China

## Abstract

Hyperphosphorylation of the microtubule-associated protein, tau, is critical to the progression of Alzheimer's disease (AD). Fuzheng Quxie Decoction (FQD), a Chinese herbal complex, is an effective clinical formula used to treat AD. In the current study, we employed high-performance liquid chromatography and liquid chromatography tandem mass spectrometry to identify the components of FQD. Three major components (ginsenoside Rg1, ginsenoside Re, and coptisine) were detected in the brain of FQD-fed mice, indicating their ability to cross the blood-brain barrier. We further evaluated the efficacy of FQD on Senescence-Accelerated Mice Prone-8 (SAMP8) mice. FQD significantly ameliorated learning and memory deficits in SAMP8 mice on the Morris Water Maze, decreasing escape latency (*p* < 0.01) and increasing swim time within the original platform-containing quadrant (*p* < 0.05). Further, FQD increased the number of neurons and intraneuronal Nissl bodies in the hippocampal CA1 region. FQD also decreased the expression of phosphorylated tau protein and increased the expression of protein phosphatase 2A (PP2A) and the N-methyl-D-aspartate receptor subunit, NR2A (*p* < 0.01). Our results indicate that FQD improves the learning and memory ability of SAMP8 mice. Moreover, our findings suggest that the protective effect of FQD is likely mediated through an inhibition of hippocampal tau hyperphosphorylation via NMDAR/PP2A-associated proteins.

## 1. Introduction

Alzheimer's disease (AD) is a progressive neurodegenerative disease, characterized by memory loss, cognitive dysfunction, and decreased quality of life. Intraneuronal neurofibrillary tangles caused by hyperphosphorylation of the microtubule-associated tau protein and extraneuronal plaques composed of amyloid-*β* (A*β*) protein are the main pathological hallmarks of AD. Due to the failure of immunotherapy against A*β* to effectively treat individuals with AD, recent research has focused on developing interventions to target aberrant phosphorylation of tau protein [1].

Tau is the main microtubule-associated protein in neurons. Normal phosphorylation of tau is required for microtubule assembly and binding activity; therefore, this protein is crucial in maintaining physiological function [2]. Hyperphosphorylation of tau protein leads to partial or complete loss of its biological activity [3]. AD pathophysiology is characterized by aggregates of abnormally phosphorylated tau in the entorhinal cortex, which spread to the hippocampus and entire cerebral cortex. The progression of this irregular protein diffusion is correlated with a decrease in cognitive function in AD [4]; thus, tau hyperphosphorylation is the central link of AD toxic effects. Both the National Institute on Aging and Alzheimer's Association (NIA-AA; 2011) and the International Working Group (IWG-2; 2014) have proposed using tau protein and p-tau protein levels in cerebrospinal fluid as a diagnostic biomarker of AD [5]. In terms of molecular mechanisms leading to the hyperphosphorylation of tau protein, both the upregulation of protein kinases and downregulation of protein phosphatases have been implicated [6]. In the human brain, PP2A is the major tau phosphatase and has been reported to account for 71% of the total tau phosphatase activity [7]. PP2A inactivation prevents tau dephosphorylation and promotes activation of protein kinase A [8].

Fuzheng Quxie Decoction (FQD) is a clinical traditional herbal formula composed of Ginseng Radix et Rhizoma (Renshen), Rhizoma Coptidis (Huanglian), and Rhizoma Ligustici Chuanxiong (Chuanxiong). Numerous studies have shown that biocomponents of FQD, including the Rg1 and Rb1 ginsenosides of Renshen, berberine of Huanglian, and ligustilide of Chuanxiong, can ameliorate learning and memory impairments in models of AD [9–15]. In this study, we aimed to investigate the effect of early FQD administration on learning and memory in Senescence-Accelerated Mice Prone-8 (SAMP8) and its regulation on tau hyperphosphorylation in the hippocampus.

## 2. Materials and Methods

### 2.1. Animals

The present study employed SAMP8 mice, which exhibit age-related cognitive dysfunction accompanied by A*β* and tau protein abnormalities, as a mouse model of AD. Senescence-resistant-1 (SAMR1) mice were used as controls. Mice in both groups were 3-month-old males, weighing 26–30 g, reared under specific pathogen-free conditions and obtained from the Department of Experimental Animal Science, Peking University Health Science Center, permit number, SCXK (Jing) 2011-0012. Throughout the experiments, animals were housed in a controlled environment, with a 12-h light/dark cycle; temperature, 23 ± 2°C; relative humidity, 50–70%. Animals received food and water ad libitum, and mice were allowed to adapt to their environment for 1 week before experiment onset.

### 2.2. Drug Preparation

FQD, composed of Renshen, Huanglian, and Chuanxiong, with a weight ratio of 9 : 6 : 5 (Table 1), was prepared into an extract by the Department of Pharmaceutical Preparation of Xiyuan Hospital, China Academy of Chinese Medical Sciences. One gram of FQD extract was equivalent to 3.71 g of crude drug. High-performance liquid chromatography (HPLC) was used to determine the main components of FQD, employing a combination of qualitative and quantitative methods for quality control. During the study, the extract was mixed with varying ratios of distilled water, resulting in concentrations of 0.7 g/ml and 3.5 g/ml (extract).

Memantine hydrochloride tablets (Ebixa 10 mg, batch number: H20120268) produced by H. Lundbeck A/S were suspended in distilled water, resulting in a concentration of 2.6 mg/ml.

### 2.3. Reagents

Rabbit monoclonal anti-Tau s396 phosphorylated antibody, mouse monoclonal anti-tau antibody (TAU-5), rabbit monoclonal anti-PP2A*α* Y307 phosphorylated antibody, rabbit monoclonal anti-N-methyl-D-aspartate receptor (NMDAR) 2A antibody, and mouse monoclonal anti-NMDAR2B antibody were acquired from Abcam (Cambridge, GRB). Rabbit anti-PP2Ac subunit antibody was purchased from Neuron Signaling Technology (Massachusetts, USA). Beta-actin mouse monoclonal antibody was provided by ImmunoWay (California, USA). Goat anti-rabbit IgG (H+L)-HRP and goat anti-mouse IgG (H+L)-HRP were provided by Beijing TDY Biotech (Beijing, CHN). BCA protein quantification kit, Coomassie Brilliant Blue staining solution, and ponceau red staining solution were provided by the Beijing Sinoble Biotechnology Center. Protease and phosphatase inhibitors were provided by Roche (Basel, CHE).

### 2.4. Animal Groups and Drug Administration

Forty male SAMP8 mice were randomly divided into four groups by a computer-generated random number table. Groups included model (equal volume distilled water), memantine (2.6 mg/kg/d), FQD low-dose (FQD-L, 0.7 g/kg/d, extract), and FQD high-dose (FQD-H, 3.5 g/kg/d, extract) groups. The control group comprised 10 male SAMR1 mice with the same genetic background as experimental mice, which were fed with distilled water. Each group received intragastric 0.1 ml/10 g/d distilled water or liquid-suspended memantine or FQD once a day for 12 weeks. All efforts were made to minimize the number of animals used and their suffering during this study. Animal procedures were performed according to the Guide for the Care and Use of Laboratory Animals and the Beijing Laboratory Animal Welfare Ethics Review Guidelines issued by the Ministry of Science and Technology of China. This study was approved by the Ethics Committee of Xiyuan Hospital, China Academy of Chinese Medical Sciences (Permit Number: CACMS/20141220X21).

### 2.5. Morris Water Maze

After 3 months of intragastric water or drug administration, spatial learning and memory were assessed using the Morris Water Maze (MWM) video analysis system (Beijing ZS Dichuang Technology Development Co., Ltd., Beijing, China). The apparatus consisted of a white pool (120 cm in diameter and 50 cm in height), platform (10 cm in diameter and 50 cm in height), video camera, and computer. The pool was divided into four quadrants, with the visible cues outside the pool. The pool was filled with opaque water, maintained at 19°C [16]. The platform was fixed in one quadrant of the pool, 1 cm below the surface of the water, and the test environment was kept quiet with a stable light source. One day before the experiment, mice were habituated to the pool environment for 120 s without the platform. The MWM experiment consisted of two parts: navigation trials and a spatial probe trial. The trials were conducted between 9:00 and 10:30 every morning to exclude any impact of circadian rhythm.

In the navigation trials, mice were placed into the water facing the wall of the pool at the 1/2 radian position in one of the four quadrants. Mice were allowed 60 s to find and mount the platform, where they remained for at least 5 s. If mice failed to find the platform within 60 s, they were guided to the platform, where they remained for 10 s before being removed, dried, and returned to their home cages. During the 5-day training period, mice underwent four trials per day, with an intertrial interval of 15 min. Time to find the platform (escape latency, EL) and search strategies were recorded using video tracking software. The maximum value for escape latency was regarded as 60 s. Search strategies were classified as spatial strategies, nonspatial strategies, or repetitive looping paths [17].

The spatial probe trial was performed on the 6th day. During this trial, the platform was removed from the pool, and mice were placed into the water facing the pool wall. Mice were allowed to swim freely for 60 s. The number of times mice crossed the original platform location, as well as total swimming time and distance in the original platform quadrant, was recorded.

### 2.6. Sample Preparation

Mice were fasted overnight after the MWM test. Five mice were randomly selected from each group and anesthetized with chloral hydrate (0.9 mL/100 g body weight). Blood samples from the retroorbital sinus were collected in heparin-treated EP tubes and centrifuged (3000 r/min, 10 min). Plasma was then collected from the samples and stored at –80°C. Next, mice were transcardially perfused with ice-cold saline, and brains were quickly removed and sagittally cut into left and right hemispheres on blocks of ice. The left half of the brain was fixed in 10% neutral formaldehyde and paraffin-embedded for pathological and immunohistochemical staining. The right half of the brain was dissected into the hippocampus and cortex. Hippocampal tissue was weighed and stored in liquid nitrogen for protein immunoblotting, while the right cerebral cortex was homogenized in 0.15 : 1 (wt/vol) ice-cold saline solution. The homogenate was centrifuged (2000 r/min, 15 min) and the supernatant was used to measure the content of brain tissue.

### 2.7. Liquid Chromatography Tandem Mass Spectrometry

Solid phase extraction (SPE) was used to prepare the plasma sample, while the acetonitrile precipitation method was used to prepare supernatant obtained from the above described method brain tissue. We then employed liquid chromatography tandem mass spectrometry (LC-MS/MS) to identity the individual components separated out by LC and to establish a standard calibration curve. If samples exhibited a good fit with the standard curve, they were determined to be accurately quantified.

### 2.8. Hematoxylin Eosin Staining

Fixed, paraffin-embedded brain tissue was sectioned and underwent hematoxylin eosin (HE) staining according to the following procedure. Sections were deparaffinized, washed in distilled water, and incubated in hematoxylin solution for 5 min; excess hematoxylin solution was washed off with running tap water. To remove background staining, sections underwent a differentiation step in hydrochloric acid alcohol, after which they were fully washed in running tap water. Sections were then counterstained in eosin solution for 2-3 min, washed in running tap water, dehydrated through graded alcohol, and mounted with neutral resin. Pathological changes in neurons were observed at 400x under a light microscope.

### 2.9. Nissl Staining

Paraffin-embedded, fixed brain tissue was deparaffinized, washed 1-2 min in distilled water, dipped in 1% thionin lyosol at 37°C for 30 min, and washed again for 1-2 min in distilled water. In order to moderately differentiate the nucleus, sections were incubated in 0.5% hydrochloric acid alcohol, washed back to blue, and differentiated using 95% alcohol until the Nissl substance was visualized. Sections were then dehydrated (twice for 5 min each) in 100% anhydrous alcohol, permeabilized with xylene twice (5 min each), and mounted with neutral gum. Changes observed in neurons and Nissl bodies of the hippocampal CA1 region were detected at 400x under a light microscope.

### 2.10. Immunohistochemistry

Sections were deparaffinized and incubated in a 3% H_2_O_2_-deionized water solution for 5–10 min to block endogenous peroxidase activity. Sections were then washed three times (2 min each) in phosphate-buffered saline (PBS), and incubated in primary antibodies overnight at 4°C. The next day, sections were washed in PBS three times (3 min each), incubated 20 min in secondary antibodies, and again washed in PBS three times (3 min each). Sections were then stained using 3,3′-diaminobenzidine, dehydrated in graded alcohol, cleared in xylene, and mounted with neutral gum. The CA1 region of the hippocampus in three adjacent fields on each section was viewed under DpxView Pro, a light microscope (DeltaPix, DEN). Tau-5 and p-tau-positive neurons were counted with Image-Pro Plus 6.0 image processing software using mean optical density (MOD). All evaluations were done by one researcher that was blind to the experimental design.

### 2.11. Western Blot Analysis

Hippocampal tissue samples (5/group) were lysed with RIPA buffer to extract protein and centrifuged at 13,000*g* for 20 min at 4°C (3-18K, Sigma). The supernatant was collected, and total protein was quantified using a BCA kit (Beijing Sinoble Biotechnology Center, Beijing, China). The protein concentration was adjusted to 4 mg/ml with RIPA and 5x reduced sample buffer and boiled for 5 min. According to the molecular weight of the target protein, we prepared either 8% or 12% acrylamide resolving gels, with the concentration of the stacking gel remaining at 5%. Samples containing 20 *μ*g of protein were loaded on a sodium dodecyl sulfate-polyacrylamide gel (SDS-PAGE) electrophoresis (90-V for the stacking gel ~20 min; 160 V for the separating gel, timed in accordance with the protein marker) and transferred to 0.45 *μ*m nitrocellulose filter membranes (300 mA, 1-2 h). Membranes were blocked with 3% bovine serum albumin-tris-buffered saline for 30 min and incubated in primary antibody solution overnight at 4°C. After incubation with horseradish peroxidase-conjugated antibody anti-rabbit or anti-mouse IgG antibodies for 40 min, protein complexes were detected using enhanced chemiluminescence Western blotting detection reagents (Millipore). Band intensity was determined using Gel-Pro Analyzer 4.0 software.

### 2.12. Statistical Analysis

Values of all experiments are represented as mean ± standard error of the mean (SEM). Statistical analyses were performed using SPSS 19.0 software. A one-way analysis of variance (ANOVA) with a least significant difference test or a one-way ANOVA with post hoc Dunnett's T3 (multiple comparisons) was used to compare between-group values.* p* < 0.05 was considered statistically significant.

## 3. Results

### 3.1. Bioactive Components of FQD

**Figure 1:**
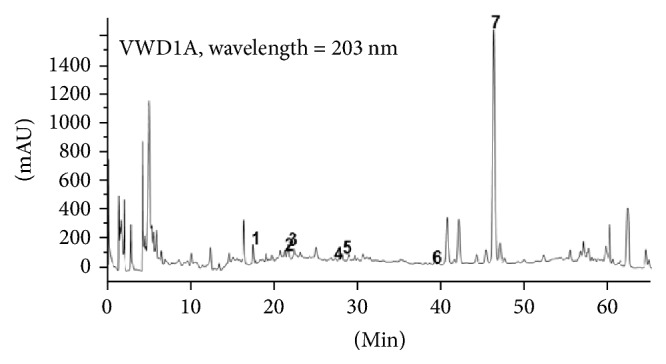
*Fingerprint map of Fuzheng Quxie Decoction (FQD) extract detected with HPLC.* 1: ferulic acid; 2: ginsenoside Rg1; 3: ginsenoside Re; 4: coptisine; 5: ginsenoside Rb1; 6: berberine; 7: ligustilide.

Using HPLC, ginsenoside Rg1 (9.86%), ginsenoside Re (2.86%), ginsenoside Rb1 (0.47%), coptisine (7.12%), berberine (2.19%), ligustilide (21.40%), and ferulic acid (0.21%) were identified as the seven main bioactive components of FQD. Their corresponding fingerprint was established using joined qualitative and quantitative methods for quality control (Figure 1).

### 3.2. Determination of FQD Components That Cross the Blood-Brain Barrier

The calibration curves of ginsenoside Rg1, ginsenoside Re, ginsenoside Rb1, coptisine, berberine, and ferulic acid in plasma and brain tissue homogenates were established using the LC-MS/MS detection method. A curve for ligustilide could not be constructed, as this component is not compatible with LC-MS/MS. Results showed a good linear relationship of the six standard curves; thus, all components could be quantified. All six components were detected in the plasma, while only ginsenosides Rg1 and Re and coptisine were detected in the brain (Table 2).

### 3.3. FQD Ameliorates Spatial Learning and Memory Deficits in SAMP8 Mice

**Figure 2:**
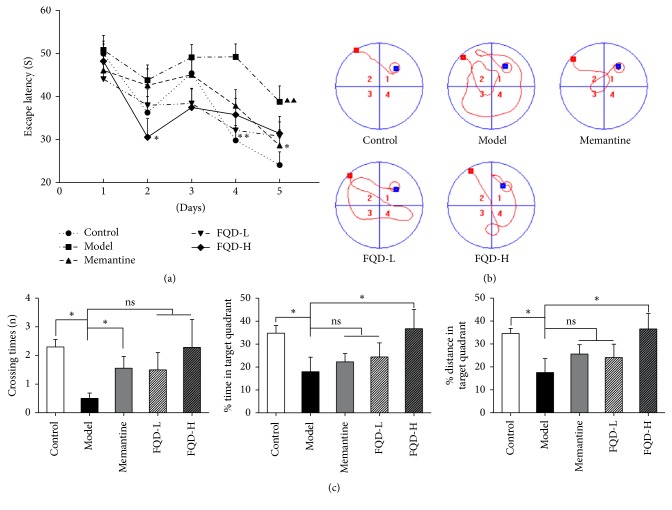
*FQD ameliorated learning and memory deficits of SAMP8 mice.* (a) All the group displayed a spatial learning effect (*p* <  0.01 or *p* <  0.05). On the last 2 days of training, the model group exhibited longer latencies to reach the platform than the control group (*p* < 0.01). Moreover, compared to the model group, memantine, FQD-L, and FQD-H groups exhibited shorter latencies to reach the platform on the fifth (*p* < 0.05), fourth (*p* < 0.01), and second (*p* < 0.05) days of training. (b) The strategies for searching platform on the fifth day of navigation trial. (c) Values representing the number of crossings, swim time, and percent distance in the target quadrant were all significantly lower in the model group compared to the control group (*p* < 0.05). Compared to the model group, the number of crossings exhibited by the memantine group and the swim time and percent distance in target quadrant of the FQD-H group were significantly higher (*p* <  0.05). No between-group difference was observed in terms of total swim distance throughout the test (*p* >  0.05). ^*∗*^*p* < 0.05, ^*∗∗*^*p* < 0.01 between model and drug-treated groups. Black triangle, *p* < 0.01 between model and control group.

On the first day of MWM testing, there was no statistically significant difference between groups in terms of escape latency (*p* > 0.05). For each group, the escape latency on the fifth day of testing was shorter than that observed on the first day (*p* < 0.01 or* p *< 0.05), indicating that all mice were able to learn. On the last 2 days of training, the model group exhibited longer latencies to reach the platform than the control group (*p* < 0.01). Moreover, when compared to the model group, memantine, FQD-L, and FQD-H groups exhibited shorter latencies to reach the platform on the fifth (*p* < 0.05), fourth (*p* < 0.01), and second (*p* < 0.05) days of training (Figure 2(a)).

Differences in search strategy may explain the observed improvements in performance during the 5 days of navigation trials. Mice of the control group utilized spatial strategies (direct: swimming directly to the platform), while those of the model group utilized nonspatial strategies (scanning: searching the interior portion of the tank without spatial bias). Performance was worse among mice of the memantine than among those of the control group. In contrast to the model group, the memantine, FQD-L, and FQD-H groups tended to adopt spatial strategies (indirect: swimming to the platform with at most one loop) (Figure 2(b)). No significant differences in performance were observed between mice of the FQD groups and those of the memantine group.

The spatial probe trial was performed on the 6th day. Values representing the number of crossings, swim time, and the percentage of the target quadrant in the total distance were significantly lower in the model group than in the control group (*p* < 0.05). Both the number of crossings exhibited by the memantine group and the swim time and the percent distance in target quadrant of the FQD-H group were significantly higher (*p *< 0.05) than the corresponding values in the model group. No between-group difference in total swim distance was observed throughout the test (*p *> 0.05) (Figure 2(c)).

### 3.4. Morphological Changes in CA1

**Figure 3:**
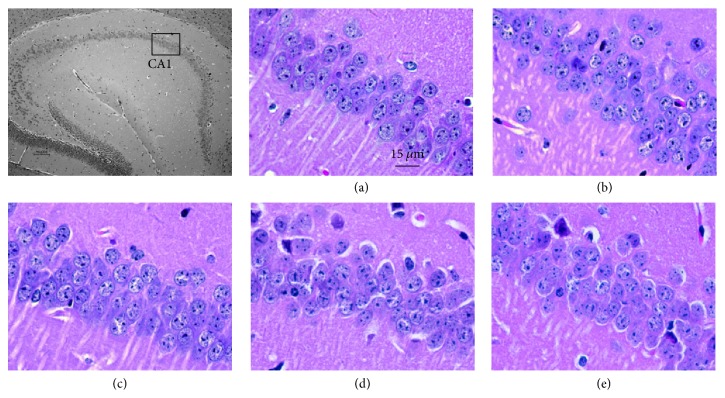
*HE staining of hippocampal CA1 (×400)*. (a) Control group. (b) Model group. (c) Memantine group. (d) FQD-L group. (E) FQD-H group.

HE staining revealed that neurons in the hippocampal CA1 region of the control group were arranged in an organized, compact pattern with clear boundaries. In the model group, however, neurons were scattered with no clear boundaries. Moreover, the number of free neurons was increased and neuron layers were decreased. The nuclei of these neurons appeared as deep pyknotic masses or, in some cases, were not even present. After 3 months of drug administration, all aspects of pathological neuron morphology were improved (Figure 3).

**Figure 4:**
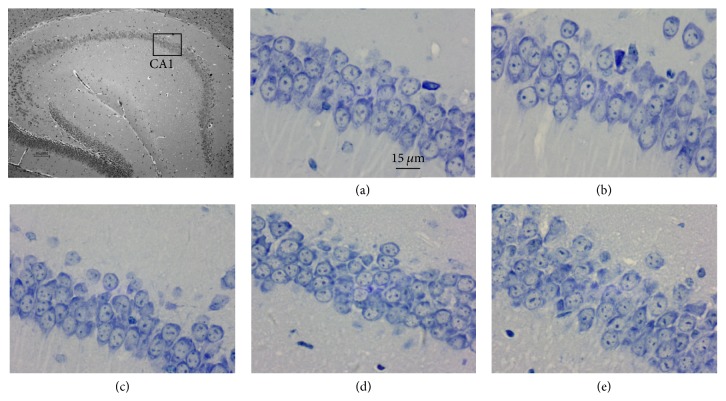
*Nissl staining of hippocampal CA1 (×400).* (a) Control group. (b) Model group. (c) Memantine group. (d) FQD-L group. (e) FQD-H group.

Nissl staining revealed that neurons in the CA1 region of the hippocampus of control mice were large in number, arranged in neat rows, and rich in Nissl bodies and exhibited pale blue nuclei and dark blue backgrounds. In contrast, neurons in the model group were lower in number, disordered, and swollen, lacked clear Nissl body boundaries, and exhibited light blue cytoplasm and dark blue pyknotic nuclei. Drug administration ameliorated the histopathology of the SAMP8 mice in the hippocampal CA1 region: neurons significantly increased in number, appeared neatly arranged, and exhibited a greater number of Nissl bodies following treatment (Figure 4).

### 3.5. FQD-Mediated Inhibition of Tau Hyperphosphorylation

**Figure 5:**
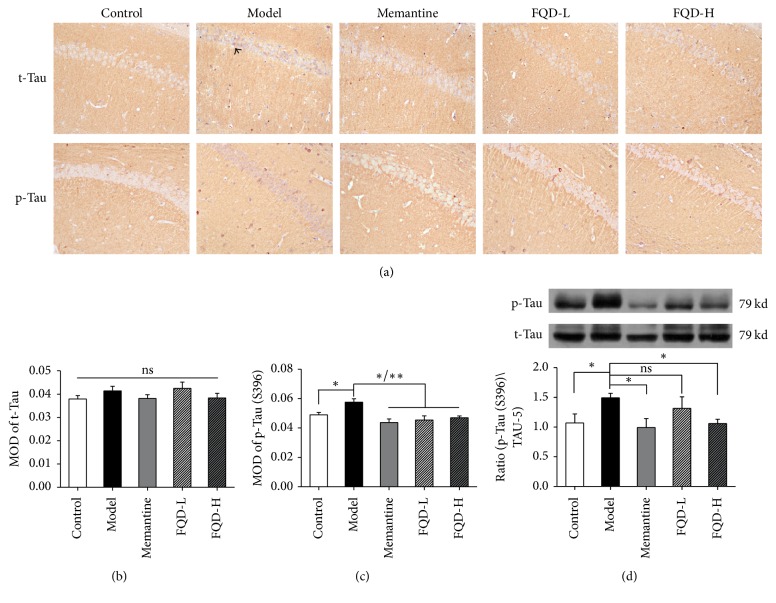
*FQD inhibited tau protein hyperphosphorylation in SAMP8 mice.* (a) Immunohistochemistry of t-Tau and p-Tau (S396) (×100). (b-c) Compared to the control group, the model group showed a significant increase in the MOD of CA1 region p-Tau (S396) (*p* < 0.05). However, the MOD of t-Tau was not increased (*p* > 0.05). After treatment with memantine and FQD, the MOD of CA1 region p-Tau (S396) was significantly decreased (*p* < 0.01 and *p* <0.05, resp.) compared to the untreated model group. The MOD of t-Tau was not statistically decreased (*p* > 0.05). (d) Western blot analyses, showing a significant increase in the level of p-Tau (S396) in model mice compared to control group (*p* < 0.05). The expression of hippocampal p-Tau (S396) was significantly decreased after administration with memantine and high doses FQD (*p* < 0.05). ^*∗*^*p* < 0.05, ^*∗∗*^*p* < 0.01.

Western blot and immunohistochemistry experiments were used to assess the expression of t-tau and p-tau at the Ser396 epitope (S396). Immunohistochemistry revealed a significant increase in the MOD of CA1 region p-Tau (S396) in the model group, relative to values observed in the control group (*p* < 0.05). However, no increases in the MOD of t-tau were observed (*p* > 0.05). After treatment with memantine or FQD, the MOD of CA1 region p-tau (S396) was significantly decreased (*p* < 0.01 and* p* < 0.05, resp.) relative to levels observed in the untreated model group. No significant decreases in the MOD of t-tau were observed (*p* > 0.05) (Figures 5(a)–5(c)).

Western blot analyses confirmed our immunohistochemistry results, revealing a significant increase in the level of p-tau (S396) in model mice relative to levels observed in control mice (*p* < 0.05). The expression of hippocampal p-tau (S396) was significantly decreased following treatment with memantine and high doses of FQD (*p* < 0.05) (Figure 5(d)).

### 3.6. Expression of NMDAR/PP2A-Associated Proteins

**Figure 6:**
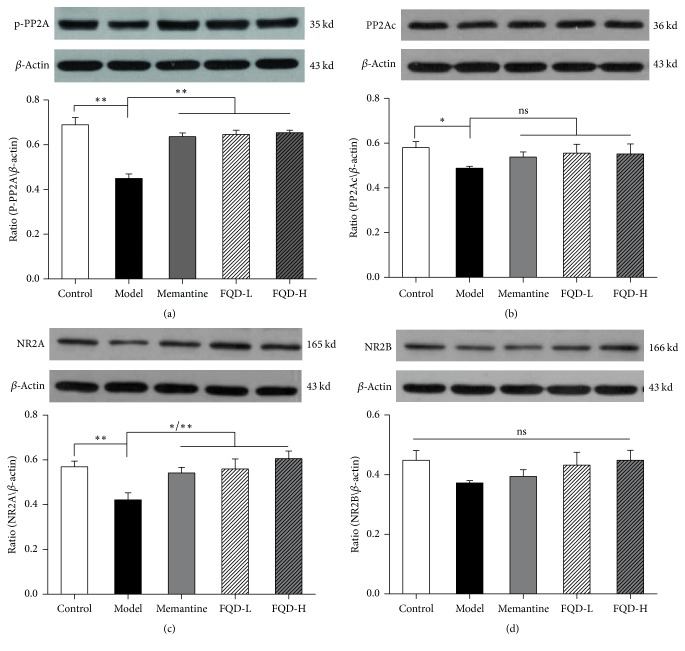
*Western blot assessed the expression of p-PP2A (a), PP2Ac (b), NR2A (c), and NR2B (d).* Compared to the control group, the expression of p-PP2A, PP2Ac, and NR2A was significantly decreased in hippocampal tissues of model group mice (*p* < 0.01 and 0.05 and *p* <0.01, resp.). However, NR2B expression was not significantly different in model mice (*p* > 0.05). The expression of hippocampal p-PP2A and NR2A was significantly elevated in drug-treated mice (*p* < 0.01); NR2A was significantly elevated in memantine and high doses of FQD treated mice (*p* < 0.01 and 0.05, resp.), while PP2Ac and NR2B were not significantly different (*p* > 0.05). ^*∗*^*p* < 0.05, ^*∗∗*^*p* < 0.01.

Western blot was used to assess the expression of p-PP2A, PP2Ac, NR2A, and NR2B. Compared to the control group, the expression of p-PP2A, PP2Ac, and NR2A was significantly decreased in hippocampal tissues of model group mice (*p* < 0.01, *p* < 0.05, and *p* < 0.01, resp.). However, NR2B expression was not significantly different in model mice (*p* > 0.05). The expression of hippocampal p-PP2A and NR2A was significantly elevated in drug-treated mice (*p* < 0.01); NR2A was significantly elevated in memantine and high doses of FQD treated mice (*p* < 0.01 and *p* < 0.05, resp.), while PP2Ac and NR2B were not significantly different (*p* > 0.05) (Figure 6).

## 4. Discussion

The findings of the present study demonstrated that FQD can ameliorate learning and memory impairments in SAMP8 mice, increase the number of intraneuronal Nissl bodies and the total number of neurons in the hippocampal CA1 region, and inhibit tau hyperphosphorylation in the hippocampus. Further, our findings indicated that the main bioactive components of FQD include ginsenosides Rg1, Re, and Rb1, as well as coptisine, berberine, ligustilide, and ferulic. We also observed that ginsenoside Rg1, ginsenoside Re, and coptisine can cross the blood-brain barrier. These findings are in agreement with those of several studies, which have revealed that the major components of FQD (i.e., ginsenosides Rg1, Rb1, and Re, as well as ferulic acid, coptisine, berberine, and ligustilide) exert antidementia effects in models of AD via one or several of the following mechanisms: reducing A*β* content [15, 18–20], inhibiting tau hyperphosphorylation [9, 11–15], reducing the occurrence of inflammatory reactions [21], regulating neurotransmitter expression and signaling [22, 23], inhibiting hippocampal glial fibrillary acidic protein expression [19], and promoting resistance to oxidative stress [23].

As SAMP8 mice exhibit rapid senescence, they are commonly used as an animal model of AD. The rapid senescence displayed by SAMP8 can be seen at 4 months, with the total lifespan of these mice limited to approximately 12-13 months [24]. SAMP8 mice are characterized by an age-related spontaneous deterioration in learning and memory, similar to the symptoms associated with AD [25, 26]. Research has revealed that these age-related behavioral alterations occur as early as 4 months of age [27]. Spatial learning and memory capacity begin to decline in these mice as early as 2−4 months, as shown by impaired performance on the MWM [28, 29].

The MWM is extensively used to evaluate spatial learning and memory in rodents. In the MWM of this study, 6-month-old SAMP8 mice exhibited significantly prolonged escape latencies, as well as a reduction in the number of crossings and total time spent in the quadrant of the maze that contained the platform location during training sessions. These findings indicate that learning and memory capacity were significantly decreased in these mice, which is consistent with findings reported by Huang et al. [25, 28, 29]. After 3 months of early FQD intervention, escape latency was significantly decreased and swim time in the original platform-containing quadrant was significantly increased, which indicates that FQD improved learning and spatial memory capacity in SAMP8 mice.

Tau hyperphosphorylation is critical in AD pathology [30]. In SAMP8 mice, tau phosphorylation at Ser202 and Ser396 has been observed in the hippocampus at 3 months of age [31]. In contrast, phosphorylation at Ser422 has been reported in the cortex of these animals at 5 months of age [32], with abnormal phosphorylation of tau appearing at 11 months [33]. Canudas et al. [34] reported that, compared to 5-month-old SAMRl mice, tau hyperphosphorylation in the cortex, striatum, and hippocampus can be found in age-matched SAMP8 mice. In the present study, levels of hippocampal p-tau (S396) were significantly increased in 6-month-old SAMP8 mice relative to those observed in SAMR1 mice, consistent with the aforementioned findings. Our results further indicated that these increases in p-Tau (S396) were inhibited following FQD or memantine intervention. Thus, higher doses of FQD may promote greater protein dephosphorylation than lower doses.

The entorhinal cortex and CA1 region of the hippocampus correspond to the first two brain regions in which AD pathological changes occur. Indeed, both regions exhibit significant reductions in neurons at the early stages of the disease, resulting in impaired spatial memory [35]. Nissl bodies are characteristic structures in a neuron's cytoplasm, serving as structural proteins that synthesize the organelles and enzymes required for neurotransmitter production. In this study, 6-month-old SAMP8 mice displayed a significant reduction in the number of neurons and intraneuronal Nissl bodies located in the CA1 region of the hippocampus; such morphological changes indicate neuronal degeneration and impaired function and are in agreement with the results of Sureda et al. [32]. After 3 months of FQD and memantine intervention, the structure and quantity of neurons and intraneuronal Nissl bodies in the CA1 have improved. Thus, both FQD and memantine prevented the age-related neuronal degeneration that these mice have typically exhibited.

Activation of NMDARs is essential for synaptic plasticity and necessary for long-term potentiation (LTP) [36]. Several studies have demonstrated that localization of the major NMDAR subunits, NR2A and NR2B, correlates with opposite functions; synaptic NR2A provides neuroprotection and mediates LTP, while extrasynaptic NR2B is linked to damage-induced toxicity and long-term depression (LTD) [37–41]. PP2A is composed of structural subunit A, regulatory subunit B, and catalytic subunit C (PP2Ac). PP2Ac plays an important role in the regulation of PP2A activity [42]. Downregulating the expression or activity of PP2A phosphatase is one of the major causes of tau hyperphosphorylation [43]. Zhang et al. showed that phosphorylation of the PP2A catalytic subunit at Y307 efficiently inactivates PP2A* in vitro* [44]. PP2A and NMDAR can form a complex such that once NMDARs are overactivated, PP2A dissociates from the complex and gets inactivated, leading to tau hyperphosphorylation [45, 46]. Mondragón-Rodríguez et al. [47] considered that inhibition of PP2A activity leads to NMDAR overactivation and then leads to tau hyperphosphorylation. NMDAR overactivation leads tau protein toxicity, which ultimately causes apoptosis [48]. As a noncompetitive antagonist of NMDARs, memantine can act as a neuroprotective agent to inhibit NR2B overactivation and block the inhibition of PP2A activity [47].

In the present study, hippocampal levels of PP2Ac, P-PP2A, and NR2A were significantly decreased in SAMP8 mice, relative to those observed in SAMR1 mice. Following FQD or memantine intervention, both p-PP2A (Y307) dephosphorylation and decreases in NR2A were significantly inhibited. The p-PP2A expression observed in the present study was similar to those described by Amadoro et al. [49]. Specifically, Liu et al. reported that p-PP2A (Y307) was decreased in an acute hypoxia-induced AD neuron model and that an increase in PP2A activity was correlated with the amount of dephosphorylated PP2A (Y307). Another* in vitro* study by Liu et al. [50] revealed that blocking intraneuronal tyrosine protein kinase Src for 12 hours significantly reduces p-PP2A (Y307) and decreases total PP2A activity. It has been shown that the expression of NR2A and NR2B in 6-month-old SAMP8 mice is significantly lower than age-matched SAMR1 [51]. This study showed that abnormal function and expression of synaptic NMDARs led to the inhibition of PP2A expression, enhancing tau phosphorylation and inhibiting dephosphorylation. These mechanisms in turn caused synaptic abnormalities and tau hyperphosphorylation in mouse hippocampal neurons, thereby affecting learning and memory capacity. We further found that FQD administration can regulate the expression of NMDAR/PP2A-associated proteins to delay neurodegenerative disease.

In conclusion, our findings demonstrated that FQD may exert protective effects against learning and memory impairments in SAMP8 mice via inhibition of tau hyperphosphorylation in the hippocampus. Although the mechanisms underlying the effects of FQD in AD models remain to be fully elucidated, our findings provide a solid foundation for future investigation of the role of tau protein phosphorylation in AD progression. As such, the traditional Chinese medicine FQD is a promising candidate for the development of novel AD treatments.

## Figures and Tables

**Table 1:** Composition of the Chinese herbal formula FQD.

Pinyin name	Source plants	Latin name	Amount/weight ratio
Renshen	*Panax ginseng* C. A. Mey.	*Ginseng Radix et Rhizoma*	9
Huanglian	*Coptis chinensis *Franch.	*Rhizoma Coptidis*	6
Chuanxiong	*Ligusticum chuanxiong*Hort.	*Rhizoma Ligustici Chuanxiong*	5

**Table 2:** FQD components detected via LC-MS/MS (ng/mL).

Component	Plasma level	Brain level
Ginseng		
Ginsenoside Rg1	2.63	1.58
Ginsenoside Re	2.63	4.47
Ginsenoside Rb1	10.7~282	ND
Huanglian		
Coptisine	0.793~94.2	0.767~3.3
Berberine	9.54~13.9	<LLOQ
Chuanxiong		
Ferulic acid	0.36~22.5	ND

*Note.* ND, not detected; <LLOQ, lower limit of quantitation. Ginsenoside Rg1 and ginsenoside Re were detected in one plasma sample and one brain tissue homogenate sample.

## References

[1] Giacobini E., Gold G. (2013). Alzheimer disease therapy-moving from amyloid-*β* to tau. *Nat Rev Neuro*.

[2] Kolarova M., García-Sierra F., Bartos A., Ricny J., Ripova D. (2012). Structure and pathology of tau protein in Alzheimer disease. *International Journal of Alzheimer's Disease*.

[3] Alonso A. D. C., Zaidi T., Grundke-Iqbal I., Iqbal K. (1994). Role of abnormally phosphorylated tau in the breakdown of microtubules in Alzheimer disease. *Proceedings of the National Acadamy of Sciences of the United States of America*.

[4] Holzer M., Holzapfel H.-P., Zedlick D., Brückner M. K., Arendt T. (1994). Abnormally phosphorylated tau protein in Alzheimer's disease: Heterogeneity of individual regional distribution and relationship to clinical severity. *Neuroscience*.

[5] Jack C. R., Knopman D. S., Weigand S. D. (2012). An operational approach to National Institute on AgingAlzheimers association criteria for preclinical Alzheimer disease. *Ann Neurol*.

[6] Buée L., Bussière T., Buée-Scherrer V., Delacourte A., Hof P. R. (2000). Tau protein isoforms, phosphorylation and role in neurodegenerative disorders. *Brain Research Reviews*.

[7] Liu F., Grundke-Iqbal I., Iqbal K., Gong C.-X. (2005). Contributions of protein phosphatases PP1, PP2A, PP2B and PP5 to the regulation of tau phosphorylation. *European Journal of Neuroscience*.

[8] Zhang Y., Ma R.-H., Li X.-C. (2014). Silencing I2PP2A rescues tau pathologies and memory deficits through rescuing PP2A and inhibiting GSK-3ß signaling in human tau transgenic mice. *Frontiers in Aging Neuroscience*.

[9] Li Y. B., Zhao X. Q., Jiang Y. H., Chen D., Wang S. L. (2013). Study on molecular target promoting human neural stem cells of ginsenoside Rg1 by gene chip. *China Journal of Chinese*.

[10] Lin Z. Y., Chen L. M., Zhang J., Pan X. D., Zhu Y. G., Ye Q. Y. (2012). a) Ginsenoside Rb1 selectively inhibits the activity of L-type voltage-gated calcium channels in cultured rat hippocampal neurons. *Acta Pharmacologica Sinica*.

[11] He Y., Zhao H., Su G. (2014). Ginsenoside Rg1 decreases neurofibrillary tangles accumulation in retina by regulating activities of neprilysin and PKA in retinal cells of ad mice model. *Journal of Molecular Neuroscience*.

[12] Durairajan S. S. K., Liu L.-F., Lu J.-H. (2012). Berberine ameliorates *β*-amyloid pathology, gliosis, and cognitive impairment in an Alzheimer's disease transgenic mouse model. *Neurobiology of Aging*.

[13] Zhao H.-H., Di J., Liu W.-S., Liu H.-L., Lai H., Lü Y.-L. (2013). Involvement of GSK3 and PP2A in ginsenoside Rb1's attenuation of aluminum-induced tau hyperphosphorylation. *Behavioural Brain Research*.

[14] Yu G., Li Y., Tian Q. (2011). Berberine attenuates calyculin A-induced cytotoxicity and Tau hyperphosphorylation in HEK293 cells. *J Alzheimers Dis*.

[15] Kuang X., Chen Y. S., Wang L. F. (2014). Klotho upregulation contributes to the neuroprotection of ligustilide in an Alzheimers disease mouse model. *Neurobiol Aging*.

[16] Sun X. L., Tian S. P., Chen Q. S. (2001). Mechanism of aging stimulated aging. *Chinese Journal of Behavioral Medical Science*.

[17] Brody D. L., Holtzman D. M. (2006). Morris water maze search strategy analysis in PDAPP mice before and after experimental traumatic brain injury. *Experimental Neurology*.

[18] Shi Y.-Q., Huang T.-W., Chen L.-M. (2010). Ginsenoside Rg1 attenuates amyloid-*β* content, regulates PKA/CREB activity, and improves cognitive performance in SAMP8 mice. *Journal of Alzheimer's Disease*.

[19] Jin B. B., Chen Q., Chen Q. L. (2011). Effects of ferulic acid on learning and memory and expression of glial fibrillary acidic in hippocampus of mice with dementia. *Acta Laser Biology Sinica*.

[20] Zhu Z. Y., He X. J., Yang Q. (2015). Effect of IDO inhibitor Coptisine on Apoptosis of PC12 Cells Induced by Combination of A*β* and IFN-*γ*. *Fudan Univ J Med Sci*.

[21] Lee K. W., Jung S. Y., Choi S. M. (2012). Effects of ginsenoside Re on LPS-induced inflammatory meditors in BV2 microglial cells. *BMC Complement Altern Med*.

[22] Kim M. S., JM Yu., Kin H. J. (2014). Ginsenoside Re and Rd enhance the exression of cholinergic markers and neuronal differentiation in Neuro-2a cells. *Biol Pharm Bull*.

[23] Huang M., Chen S., Liang Y. (2016). The Role of Berberine in the Multi-Target Treatment of Senile Dementia. *Curr Top Med Chem*.

[24] Tomobe K., Nomura Y. (2009). Neurochemistry, neuropathology, and heredity in samp8: A mouse model of senescence. *Neurochemical Research*.

[25] Nitta A., Naruhashi K., Umemura M. (1995). Age-related changes in learning and memory and cholinergic neuronal function in senescence accelerated mice (SAM). *Behavioural Brain Research*.

[26] Pallas M., Camins A., Smith M. A., Perry G., Lee H.-G., Casadesus G. (2008). From aging to Alzheimer's disease: unveiling ‘The switch’ with the senescence-accelerated mouse model (SAMP8). *Journal of Alzheimer's Disease*.

[27] Yanai S., Endo S. (2016). Early onset of behavioral alterations in senescence-accelerated mouse prone 8 (SAMP8). *Behavioural Brain Research*.

[28] Huang Y., Hu Z., Liu G., Zhou W., Zhang Y. (2013). Cytokines induced by long-term potentiation (LTP) recording: A potential explanation for the lack of correspondence between learning/memory performance and LTP. *Neuroscience*.

[29] Cheng H., Yu J., Jiang Z. (2008). Acupuncture improves cognitivedeficits and regulates the brain cell proliferation of SAMP8 mice. *Neurosci Lett*.

[30] Alafuzoff I., Arzberger T., Al-Sarraj S. (2008). Staging of neurofibrillary pathology in Alzheimer's disease: a study of the BrainNet Europe Consortium. *Brain Pathol*.

[31] Tajes M., Gutierrez-Cuesta J., Folch J. (2008). Lithium treatment decreases activities of tau kinases in a murine model of senescence. *J Neuropathol Exp Neurol*.

[32] Sureda F. X., Gutierrez-Cuesta J., Romeu M. (2006). Changes in oxidative stress parameters and neurodegeneration markers in the brain of the senescence-accelerated mice SAMP-8. *Experimental Gerontology*.

[33] Wei X., Zhang Y., Zhou J. (1999). Alzheimers disease-related gene emprssion in the brain of senescerce accelerated mouse. *Neurosci Lett*.

[34] Canudas A. M., Gutierrez-Cuesta J., Rodríguez M. I. (2005). Hyperphosphorylation of microtubule-associated protein tau in senescence-accelerated mouse (SAM). *Mechanisms of Ageing and Development*.

[35] Squire L. R., kandel Eric R., Hong L. (2014). *Perspective Memory*.

[36] Howard E., Zhou R. L., Guo X. Y., Ye M. L. (2008). *Cognitive Neuroscience of Memory: An Introduction*.

[37] Charton J. P., Herkert M., Becker C.-M., Schröder H. (1999). Cellular and subcellular localization of the 2B-subunit of the NMDA receptor in the adult rat telencephalon. *Brain Research*.

[38] Chen M., Lu T.-J., Chen X.-J. (2008). Differential roles of NMDA receptor subtypes in ischemic neuronal cell death and ischemic tolerance. *Stroke*.

[39] Hardingham G. E., Bading H. (2010). Synaptic versus extrasynaptic NMDA receptor signalling: implications for neurodegenerative disorders. *Nature Reviews Neuroscience*.

[40] Bordji K., Becerril-Ortega J., Nicole O., Buisson A. (2010). Activation of extrasynaptic, but not synaptic, NMDA receptors modifies amyloid precursor protein expression pattern and increases amyloid-*β* production. *The Journal of Neuroscience*.

[41] Collingridge G. L., Isaac J. T. R., Yu T. W. (2004). Receptor trafficking and synaptic plasticity. *Nature Reviews Neuroscience*.

[42] L. Collingridge G., T. R. Isaac J., Tian Wang Y. (2004). Receptor trafficking and synaptic plasticity. *Reviews Neuroscience*.

[43] Bryant J. C., Westphal R. S., Wadzinski B. E. (1999). Methylated C-terminal leucine residue of PP2A catalytic subunit is important for binding of regulatory B*α* subunit. *Biochemical Journal*.

[44] Zhang C. E., Tian Q., Wei W. (2008). Homocysteine induces tau hyperphosphorylation by inactivating protein phosphatase-2A. *Neurobiol Aging*.

[45] Chen J., Martin B. L., Brautigan D. L. (1992). Regulation of protein serine-threonine phosphatase type-2A by tyrosine phosphorylation. *Science*.

[46] Chan S. F., Sucher N. J. (2001). An NMDA receptor signaling complex with protein phosphatase 2A. *Journal of Neuroscience*.

[47] Mondragón-Rodríguez S., Trillaud-Doppia E., Dudilot A. (2012). Interaction of endogenous tau protein with synaptic proteins is regulated by N-methyl-D-aspartate receptor-dependent tau phosphorylation. *The Journal of Biological Chemistry*.

[48] Zimmer E. R., Leuzy A., Souza D. O., Portela L. V. (2016). Inhibition of Protein Phosphatase 2A: focus on the Glutamatergic System. *Molecular Neurobiology*.

[49] Amadoro G., Ciotti M. T., Costanzi M., Cestari V., Calissano P., Canu N. (2006). NMDA receptor mediates tau-induced neurotoxicity by calpain and ERK/MAPK activation. *Proceedings of the National Acadamy of Sciences of the United States of America*.

[50] Liu R., Pei J.-J., Wang X.-C. (2005). Acute anoxia induces tau dephosphorylation in rat brain slices and its possible underlying mechanisms. *Journal of Neurochemistry*.

[51] Liu R., Zhou X. W., Wang J. Z. (2007). Effects of Blocking Tyrosine Protein Kinase Src on Protein Phosphatase 2A Activity and Tau Protein Phosphorylation. *Neural Injury and Functional Reconstruction*.

